# 7-(1,3-Dioxolan-2-ylmethyl)-1,3-di­methyl-2,6-dioxo-2,3,6,7-tetra­hydro-1*H*-purin-9-ium tetra­chloridoferrate(III)

**DOI:** 10.1107/S1600536810034720

**Published:** 2010-09-11

**Authors:** Ping Ping Wang, Jiang Gong, Shi-Feng Ni

**Affiliations:** a College of Pharmaceutical Sciences, Zhejiang University of Technology, Hangzhou 310014, People’s Republic of China; b Department of Medicine, Tibet Nationalities Institute, Xianyang, Shaanxi 712082, People’s Republic of China; c Key Laboratory of Resource Biology and Biotechnology in Western China, Ministry of Education, College of Life Science, Northwest University, Xi’an 710069, People’s Republic of China

## Abstract

The asymmetric unit of the title compound, (C_11_H_15_N_4_O_4_)[FeCl_4_], contains two independent protonated 7-(1,3-dioxolan-2-ylmeth­yl)-3,7-dihydro-1,3-dimethyl-1*H*-purine-2,6-dione (doxofyllinium) and two tetrahedral tetra­chlorido­ferrate(III) anions. In the doxofyllinium, two disordered methyl­ene C atoms are observed in each dioxolane ring with an occupancy ratio of 0.54 (4):0.46 (4). In the crystal, mol­ecules are connected by N—H⋯O hydrogen bonds and weak C—H⋯O and C—H⋯Cl inter­actions.

## Related literature

For the biological activity of the drug doxofylline, see: Franzone *et al.* (1981 ▶, 1989 ▶); Zhao & Li (2001 ▶). For bond distances and angles in other tetra­chlorido­ferrate(III) compounds, see: Barbaro *et al.* (1992 ▶); Bottomley *et al.* (1984 ▶). For the synthesis of doxofylline, see: Li *et al.* (1995 ▶).
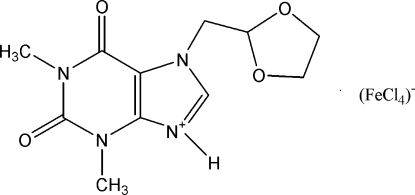


## Experimental

### 

#### Crystal data

(C_11_H_15_N_4_O_4_)[FeCl_4_]
*M* *_r_* = 464.92
Tetragonal, 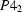

*a* = 20.2947 (4) Å
*c* = 9.0692 (4) Å
*V* = 3735.38 (19) Å^3^
*Z* = 8
Mo *K*α radiation
μ = 1.40 mm^−1^
*T* = 293 K
0.27 × 0.16 × 0.13 mm

#### Data collection

Bruker SMART APEX area-detector diffractometer
Absorption correction: multi-scan (*SADABS*; Bruker, 2000 ▶) *T*_min_ = 0.775, *T*_max_ = 0.852
19874 measured reflections
6253 independent reflections
5589 reflections with *I* > 2σ(*I*)
*R*_int_ = 0.046

#### Refinement

*R*[*F* ^2^ > 2σ(*F* ^2^)] = 0.099
*wR*(*F* ^2^) = 0.207
*S* = 1.30
6253 reflections
447 parameters
13 restraints
H-atom parameters constrained
Δρ_max_ = 0.49 e Å^−3^
Δρ_min_ = −0.31 e Å^−3^
Absolute structure: Flack (1983 ▶), 2724 Friedel pairs
Flack parameter: 0.10 (4)

### 

Data collection: *SMART* (Bruker, 2000 ▶); cell refinement: *SAINT* (Bruker, 2000 ▶); data reduction: *SAINT*; program(s) used to solve structure: *SHELXS97* (Sheldrick, 2008 ▶); program(s) used to refine structure: *SHELXL97* (Sheldrick, 2008 ▶); molecular graphics: *SHELXTL* (Sheldrick, 2008 ▶); software used to prepare material for publication: *SHELXL97*.

## Supplementary Material

Crystal structure: contains datablocks global, I. DOI: 10.1107/S1600536810034720/jh2188sup1.cif

Structure factors: contains datablocks I. DOI: 10.1107/S1600536810034720/jh2188Isup2.hkl

Additional supplementary materials:  crystallographic information; 3D view; checkCIF report

## Figures and Tables

**Table 1:** Hydrogen-bond geometry (Å, °)

*D*—H⋯*A*	*D*—H	H⋯*A*	*D*⋯*A*	*D*—H⋯*A*
N4—H4⋯O2^i^	0.86	1.93	2.774 (9)	165
N8—H8⋯O6^ii^	0.86	1.91	2.754 (9)	166
C5—H5⋯Cl5^i^	0.93	2.80	3.650 (11)	153
C8—H8*B*⋯O7^iii^	0.97	2.29	3.114 (11)	142
C10—H10*C*⋯O5^iv^	0.95	2.46	3.098 (11)	124
C10′—H10*B*⋯O5^iv^	0.89	2.57	3.384 (11)	153
C10′—H10*C*⋯O5^iv^	0.97	2.46	3.384 (11)	159
C16—H16⋯Cl1^iii^	0.93	2.78	3.560 (11)	143
C19—H19*A*⋯O4^v^	0.97	2.48	3.227 (11)	134
C19—H19*A*⋯O5	0.97	2.53	3.167 (11)	123
C22′—H22*D*⋯O1	0.97	2.58	3.242 (11)	126

Symmetry codes: (i) 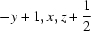; (ii) 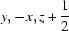; (iii) 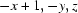; (iv) 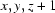; (v) 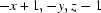.

**Fig. 1.**
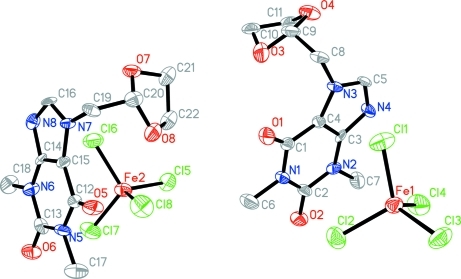
A view of the asymmetric unit of (I) with atom labels, showing 40% probability displacement ellipsoids. Parts of disorder furan rings are omitted for clarity.

## supplementary crystallographic information

### Comment

Doxofylline [7-(1,3-dioxolan-2-ylmethyl)-1,3-dimethyl-3,7- dihydro-1*H*-purine-2,6-dione] is a xanthine drug with anti-asthmatic (Franzone *et al.*, 1989), antiinflammatory (Zhao *et al.*, 2001), and bronchodilating activities (Franzone *et al.*, 1981). Now we present here the structure of the title compound, (I).

In the title compound (Fig. 1), (I), the asymmetric unit contain two crystallographically independent molecules of doxofyllinium tetrachloroferrate(III). The furan rings of the doxofyllinium are disordered, and the five atoms of these rings aren't coplanar. The iron cation is tetra coordinated by chlorine anions, and it adopts a slightly distorted tetrahedral coordination with two angles smaller than the tetrahedral one, two almost equal to tetrahedral and two larger than tetrahedral (Table 1). Fe—Cl distances spanning the range 2.172 (3) Å-2.190 (4) Å, and Cl—Fe—Cl angles 107.41 (16)°-111.77 (15)°, are similar to those found in other tetrachloroferrate(III) (Bottomley *et al.*, 1984; Barbaro *et al.*, 1992).

In the crystal, doxofyllinium cations are linked by N8—H8···O6^i^ and N4—H4···O2^ii^ hydrogen bonds (Table 2). The weak C—H···O and C—H···Cl interactions further link (I), reinforcing the structure (Table 2).

### Experimental

Doxofylline was synthesized according to Li *et al.* (1995). Doxofylline, hydrochloric acid and trichloride were dissolved in sufficient ethanol by heating to 333 K, where a yellow solution resulted. Crystals of (I) were formed by gradual evaporation of ethanol over a period of one week are 293 K.

### Refinement

All of the H atoms were placed in calculated positions and allowed to ride on their parent atoms at distances of 0.93 (C5—H5), 0.96 (methyl), 0.97 (methylene) and 0.98Å (methine), with *U*_iso_(H) = 1.2–1.5 *U*_eq_(C).

### Crystal data

**Table:**

(C_11_H_15_N_4_O_4_)[FeCl_4_]	*D*_x_ = 1.653 Mg m^−^^3^
*M**_r_* = 464.92	Mo *K*α radiation, λ = 0.71073 Å
Tetragonal, *P*4_2_	Cell parameters from 8917 reflections
Hall symbol: P 4c	θ = 2.2–26.5°
*a* = 20.2947 (4) Å	µ = 1.40 mm^−^^1^
*c* = 9.0692 (4) Å	*T* = 293 K
*V* = 3735.38 (19) Å^3^	Block, colourless
*Z* = 8	0.27 × 0.16 × 0.13 mm
*F*(000) = 1880.0	

### Data collection

**Table:**

Bruker SMART APEX area-detector diffractometer	6253 independent reflections
Radiation source: fine-focus sealed tube	5589 reflections with *I* > 2σ(*I*)
graphite	*R*_int_ = 0.046
φ and ω scan	θ_max_ = 25.0°, θ_min_ = 1.0°
Absorption correction: multi-scan (*SADABS*; Bruker, 2000)	*h* = −24→23
*T*_min_ = 0.775, *T*_max_ = 0.852	*k* = −24→23
19874 measured reflections	*l* = −9→10

### Refinement

**Table:**

Refinement on *F*^2^	Secondary atom site location: difference Fourier map
Least-squares matrix: full	Hydrogen site location: inferred from neighbouring sites
*R*[*F*^2^ > 2σ(*F*^2^)] = 0.099	H-atom parameters constrained
*wR*(*F*^2^) = 0.207	*w* = 1/[σ^2^(*F*_o_^2^) + (0.0665*P*)^2^ + 7.3566*P*] where *P* = (*F*_o_^2^ + 2*F*_c_^2^)/3
*S* = 1.30	(Δ/σ)_max_ < 0.001
6253 reflections	Δρ_max_ = 0.49 e Å^−^^3^
447 parameters	Δρ_min_ = −0.31 e Å^−^^3^
13 restraints	Absolute structure: Flack (1983), 2724 Friedel pairs
Primary atom site location: structure-invariant direct methods	Flack parameter: 0.10 (4)

### Special details

**Table:**

Geometry. All e.s.d.'s (except the e.s.d. in the dihedral angle between two l.s. planes) are estimated using the full covariance matrix. The cell e.s.d.'s are taken into account individually in the estimation of e.s.d.'s in distances, angles and torsion angles; correlations between e.s.d.'s in cell parameters are only used when they are defined by crystal symmetry. An approximate (isotropic) treatment of cell e.s.d.'s is used for estimating e.s.d.'s involving l.s. planes.
Refinement. Refinement of *F*^2^ against ALL reflections. The weighted *R*-factor *wR* and goodness of fit *S* are based on *F*^2^, conventional *R*-factors *R* are based on *F*, with *F* set to zero for negative *F*^2^. The threshold expression of *F*^2^ > σ(*F*^2^) is used only for calculating *R*-factors(gt) *etc*. and is not relevant to the choice of reflections for refinement. *R*-factors based on *F*^2^ are statistically about twice as large as those based on *F*, and *R*- factors based on ALL data will be even larger.

### Fractional atomic coordinates and isotropic or equivalent isotropic displacement parameters (Å^2^)

**Table:**

	*x*	*y*	*z*	*U*_iso_*/*U*_eq_	Occ. (<1)
Fe1	0.68089 (8)	0.33071 (7)	0.16159 (18)	0.0569 (4)	
Fe2	0.16760 (8)	0.17898 (7)	0.22058 (17)	0.0586 (4)	
Cl1	0.69861 (18)	0.24505 (16)	0.2998 (4)	0.0869 (10)	
Cl2	0.59364 (17)	0.3131 (2)	0.0264 (4)	0.0898 (11)	
Cl3	0.76550 (17)	0.35346 (18)	0.0238 (4)	0.0913 (11)	
Cl4	0.6604 (2)	0.41656 (17)	0.3005 (4)	0.0913 (11)	
Cl5	0.17996 (16)	0.25893 (15)	0.3795 (4)	0.0735 (9)	
Cl6	0.1499 (2)	0.08671 (15)	0.3379 (4)	0.0886 (10)	
Cl7	0.08268 (18)	0.20197 (19)	0.0809 (5)	0.0966 (11)	
Cl8	0.25566 (19)	0.1697 (2)	0.0873 (5)	0.1016 (12)	
O1	0.5196 (4)	0.1673 (3)	0.2934 (8)	0.0592 (19)	
O2	0.4364 (3)	0.3755 (3)	0.2618 (9)	0.063 (2)	
O3	0.4982 (4)	0.1135 (4)	0.6244 (10)	0.086 (3)	
O4	0.5648 (4)	0.1321 (4)	0.8177 (8)	0.072 (2)	
O5	0.3355 (3)	0.0243 (4)	−0.1686 (8)	0.0587 (19)	
O6	0.1189 (4)	0.0815 (4)	−0.2061 (9)	0.068 (2)	
O7	0.3705 (4)	−0.0319 (3)	0.3520 (8)	0.063 (2)	
O8	0.3889 (4)	0.0414 (4)	0.1734 (9)	0.073 (2)	
N1	0.4750 (4)	0.2704 (4)	0.2853 (9)	0.0448 (19)	
N2	0.5058 (4)	0.3546 (3)	0.4494 (9)	0.044 (2)	
N3	0.5913 (3)	0.2156 (3)	0.5737 (8)	0.0370 (16)	
N4	0.5845 (4)	0.3180 (4)	0.6382 (9)	0.0450 (19)	
H4	0.5907	0.3539	0.6865	0.054*	
N5	0.2260 (4)	0.0565 (4)	−0.1796 (9)	0.0464 (19)	
N6	0.1468 (4)	0.0128 (4)	−0.0221 (10)	0.047 (2)	
N7	0.2938 (3)	−0.0580 (3)	0.1018 (8)	0.0343 (16)	
N8	0.1903 (4)	−0.0635 (3)	0.1613 (9)	0.0452 (19)	
H8	0.1551	−0.0744	0.2081	0.054*	
C1	0.5154 (4)	0.2206 (4)	0.3469 (11)	0.040 (2)	
C2	0.4696 (4)	0.3359 (5)	0.3274 (12)	0.046 (2)	
C3	0.5443 (4)	0.3099 (3)	0.5178 (10)	0.0317 (19)	
C4	0.5473 (4)	0.2455 (4)	0.4781 (10)	0.039 (2)	
C5	0.6115 (4)	0.2604 (4)	0.6661 (11)	0.040 (2)	
H5	0.6413	0.2526	0.7420	0.048*	
C6	0.4343 (6)	0.2508 (6)	0.1589 (13)	0.068 (3)	
H6A	0.4414	0.2050	0.1380	0.103*	
H6B	0.4463	0.2766	0.0744	0.103*	
H6C	0.3887	0.2581	0.1817	0.103*	
C7	0.5043 (5)	0.4240 (5)	0.4975 (16)	0.068 (3)	
H7A	0.4743	0.4483	0.4364	0.102*	
H7B	0.5476	0.4426	0.4894	0.102*	
H7C	0.4899	0.4262	0.5983	0.102*	
C8	0.6086 (5)	0.1450 (4)	0.5793 (11)	0.049 (2)	
H8A	0.6530	0.1401	0.6176	0.059*	
H8B	0.6077	0.1268	0.4805	0.059*	
C9	0.5614 (6)	0.1078 (5)	0.6754 (12)	0.060 (3)	
H9	0.5739	0.0611	0.6762	0.072*	
C10	0.4622 (13)	0.1031 (15)	0.7592 (18)	0.068 (8)	0.46 (4)
H10A	0.4659	0.0580	0.7939	0.081*	0.46 (4)
H10B	0.4160	0.1147	0.7488	0.081*	0.46 (4)
C11	0.4989 (6)	0.1514 (14)	0.859 (3)	0.068 (8)	0.46 (4)
H11A	0.4895	0.1969	0.8345	0.081*	0.46 (4)
H11B	0.4901	0.1436	0.9627	0.081*	0.46 (4)
C10'	0.4559 (11)	0.1331 (16)	0.7455 (19)	0.094 (10)	0.54 (4)
H10C	0.4157	0.1072	0.7479	0.113*	0.54 (4)
H10D	0.4448	0.1795	0.7401	0.113*	0.54 (4)
C11'	0.4999 (7)	0.1183 (18)	0.878 (3)	0.094 (10)	0.54 (4)
H11C	0.4898	0.1469	0.9609	0.113*	0.54 (4)
H11D	0.4960	0.0727	0.9091	0.113*	0.54 (4)
C12	0.2815 (5)	0.0217 (4)	−0.1225 (11)	0.043 (2)	
C13	0.1621 (4)	0.0531 (4)	−0.1418 (12)	0.044 (2)	
C14	0.1953 (4)	−0.0211 (3)	0.0451 (10)	0.0309 (19)	
C15	0.2591 (4)	−0.0156 (4)	0.0084 (10)	0.035 (2)	
C16	0.2515 (5)	−0.0846 (4)	0.1871 (11)	0.042 (2)	
H16	0.2622	−0.1153	0.2593	0.050*	
C17	0.2421 (6)	0.1030 (6)	−0.3009 (13)	0.080 (4)	
H17A	0.2887	0.1019	−0.3196	0.119*	
H17B	0.2294	0.1468	−0.2731	0.119*	
H17C	0.2188	0.0902	−0.3885	0.119*	
C18	0.0772 (5)	0.0074 (6)	0.0225 (16)	0.071 (4)	
H18A	0.0507	0.0353	−0.0389	0.107*	
H18B	0.0726	0.0207	0.1235	0.107*	
H18C	0.0629	−0.0375	0.0119	0.107*	
C19	0.3647 (4)	−0.0690 (5)	0.1035 (11)	0.045 (2)	
H19A	0.3825	−0.0610	0.0058	0.054*	
H19B	0.3736	−0.1145	0.1292	0.054*	
C20	0.3981 (4)	−0.0246 (5)	0.2117 (13)	0.054 (3)	
H20	0.4453	−0.0347	0.2153	0.065*	
C22	0.367 (2)	0.0769 (14)	0.303 (2)	0.065 (11)	0.36 (4)
H22A	0.3207	0.0863	0.2988	0.078*	0.36 (4)
H22B	0.3915	0.1179	0.3141	0.078*	0.36 (4)
C21	0.383 (3)	0.0292 (8)	0.429 (3)	0.065 (11)	0.36 (4)
H21A	0.4289	0.0327	0.4606	0.078*	0.36 (4)
H21B	0.3543	0.0353	0.5127	0.078*	0.36 (4)
C22'	0.3901 (12)	0.0742 (11)	0.3145 (17)	0.078 (7)	0.64 (4)
H22C	0.3718	0.1183	0.3076	0.094*	0.64 (4)
H22D	0.4346	0.0769	0.3530	0.094*	0.64 (4)
C21'	0.3470 (14)	0.0299 (6)	0.411 (3)	0.078 (7)	0.64 (4)
H21C	0.3566	0.0348	0.5150	0.094*	0.64 (4)
H21D	0.3004	0.0365	0.3931	0.094*	0.64 (4)

### Atomic displacement parameters (Å^2^)

**Table:**

	*U*^11^	*U*^22^	*U*^33^	*U*^12^	*U*^13^	*U*^23^
Fe1	0.0695 (10)	0.0601 (10)	0.0411 (9)	−0.0070 (8)	0.0035 (8)	0.0009 (8)
Fe2	0.0745 (11)	0.0581 (10)	0.0432 (9)	0.0120 (8)	0.0031 (8)	0.0023 (8)
Cl1	0.105 (2)	0.078 (2)	0.078 (2)	0.0085 (18)	0.021 (2)	0.0265 (19)
Cl2	0.089 (2)	0.126 (3)	0.055 (2)	−0.026 (2)	−0.0054 (17)	−0.0144 (19)
Cl3	0.083 (2)	0.096 (2)	0.095 (3)	−0.0082 (18)	0.027 (2)	0.032 (2)
Cl4	0.128 (3)	0.082 (2)	0.064 (2)	0.003 (2)	−0.008 (2)	−0.0211 (18)
Cl5	0.087 (2)	0.0716 (18)	0.0619 (19)	−0.0058 (16)	0.0210 (17)	−0.0146 (15)
Cl6	0.136 (3)	0.0681 (19)	0.061 (2)	0.0021 (19)	−0.004 (2)	0.0153 (16)
Cl7	0.101 (3)	0.108 (3)	0.081 (2)	0.023 (2)	−0.027 (2)	0.010 (2)
Cl8	0.102 (3)	0.129 (3)	0.073 (2)	0.011 (2)	0.027 (2)	−0.018 (2)
O1	0.090 (5)	0.040 (4)	0.047 (4)	−0.004 (3)	−0.018 (4)	−0.019 (3)
O2	0.045 (4)	0.064 (4)	0.079 (6)	0.019 (3)	−0.012 (4)	0.022 (4)
O3	0.093 (6)	0.087 (6)	0.078 (7)	−0.033 (5)	−0.019 (5)	0.002 (5)
O4	0.086 (5)	0.093 (6)	0.036 (4)	0.012 (4)	−0.006 (4)	0.013 (4)
O5	0.035 (4)	0.097 (5)	0.044 (4)	−0.002 (3)	0.015 (3)	0.027 (4)
O6	0.065 (5)	0.068 (5)	0.071 (6)	0.020 (4)	−0.016 (4)	0.017 (4)
O7	0.098 (6)	0.055 (4)	0.034 (4)	−0.003 (4)	−0.015 (4)	0.011 (3)
O8	0.081 (5)	0.066 (5)	0.073 (6)	−0.024 (4)	0.002 (5)	0.009 (4)
N1	0.049 (4)	0.055 (5)	0.031 (4)	−0.003 (4)	−0.008 (4)	0.005 (4)
N2	0.050 (5)	0.024 (4)	0.056 (6)	0.008 (3)	−0.005 (4)	0.010 (3)
N3	0.048 (4)	0.041 (4)	0.022 (4)	0.009 (3)	−0.005 (3)	0.004 (3)
N4	0.061 (5)	0.036 (4)	0.038 (5)	0.003 (3)	−0.021 (4)	−0.014 (3)
N5	0.061 (5)	0.047 (4)	0.032 (4)	0.010 (4)	−0.010 (4)	0.009 (4)
N6	0.035 (4)	0.050 (5)	0.056 (5)	0.005 (3)	0.005 (4)	0.015 (4)
N7	0.037 (4)	0.039 (4)	0.027 (4)	0.003 (3)	0.002 (3)	0.012 (3)
N8	0.040 (4)	0.046 (4)	0.049 (5)	−0.010 (3)	0.013 (4)	0.000 (4)
C1	0.033 (5)	0.047 (5)	0.040 (6)	−0.003 (4)	0.004 (4)	0.010 (5)
C2	0.033 (5)	0.060 (6)	0.044 (6)	0.005 (4)	−0.009 (4)	0.014 (5)
C3	0.047 (5)	0.015 (4)	0.033 (5)	−0.012 (3)	0.009 (4)	−0.004 (3)
C4	0.038 (5)	0.052 (6)	0.026 (5)	−0.007 (4)	0.002 (4)	0.007 (4)
C5	0.052 (5)	0.035 (5)	0.033 (5)	0.004 (4)	−0.022 (4)	0.004 (4)
C6	0.088 (8)	0.071 (7)	0.046 (7)	0.008 (6)	−0.020 (6)	−0.001 (6)
C7	0.060 (7)	0.044 (6)	0.101 (10)	0.008 (5)	−0.027 (7)	0.001 (6)
C8	0.065 (6)	0.052 (6)	0.030 (5)	0.018 (5)	−0.010 (5)	0.003 (4)
C9	0.096 (9)	0.045 (6)	0.039 (6)	0.006 (5)	−0.021 (6)	0.010 (5)
C10	0.057 (13)	0.060 (14)	0.085 (17)	0.011 (9)	−0.009 (10)	0.043 (12)
C11	0.057 (13)	0.060 (14)	0.085 (17)	0.011 (9)	−0.009 (10)	0.043 (12)
C10'	0.083 (15)	0.061 (16)	0.14 (2)	0.025 (11)	0.014 (14)	0.054 (14)
C11'	0.083 (15)	0.061 (16)	0.14 (2)	0.025 (11)	0.014 (14)	0.054 (14)
C12	0.053 (6)	0.035 (5)	0.041 (6)	−0.004 (4)	−0.007 (5)	0.005 (4)
C13	0.026 (4)	0.046 (5)	0.060 (7)	0.017 (4)	−0.014 (5)	−0.009 (5)
C14	0.037 (5)	0.019 (4)	0.037 (5)	−0.008 (3)	0.006 (4)	0.007 (3)
C15	0.048 (5)	0.025 (4)	0.032 (5)	0.003 (4)	0.002 (4)	0.002 (4)
C16	0.061 (6)	0.021 (4)	0.043 (6)	0.007 (4)	−0.008 (5)	0.018 (4)
C17	0.096 (9)	0.094 (9)	0.049 (8)	0.012 (7)	0.012 (7)	0.020 (7)
C18	0.038 (6)	0.072 (7)	0.103 (10)	0.003 (5)	0.003 (6)	0.015 (7)
C19	0.035 (5)	0.057 (6)	0.042 (6)	0.019 (4)	−0.008 (4)	0.007 (5)
C20	0.034 (5)	0.059 (6)	0.069 (8)	0.011 (4)	0.003 (5)	0.015 (6)
C22	0.06 (2)	0.051 (16)	0.08 (2)	−0.008 (12)	0.016 (14)	0.002 (14)
C21	0.06 (2)	0.051 (16)	0.08 (2)	−0.008 (12)	0.016 (14)	0.002 (14)
C22'	0.063 (12)	0.064 (11)	0.108 (15)	−0.001 (7)	−0.001 (9)	−0.009 (10)
C21'	0.063 (12)	0.064 (11)	0.108 (15)	−0.001 (7)	−0.001 (9)	−0.009 (10)

### Geometric parameters (Å, °)

**Table:**

Fe1—Cl1	2.173 (3)	N8—H8	0.8600
Fe1—Cl3	2.173 (3)	C1—C4	1.446 (13)
Fe1—Cl2	2.183 (4)	C3—C4	1.357 (12)
Fe1—Cl4	2.190 (4)	C5—H5	0.9300
Fe2—Cl8	2.165 (4)	C6—H6A	0.9600
Fe2—Cl6	2.183 (3)	C6—H6B	0.9600
Fe2—Cl5	2.185 (3)	C6—H6C	0.9600
Fe2—Cl7	2.189 (4)	C7—H7A	0.9600
O1—C1	1.189 (10)	C7—H7B	0.9600
O2—C2	1.206 (11)	C7—H7C	0.9600
O3—C9	1.368 (13)	C8—C9	1.499 (14)
O3—C10	1.441 (10)	C8—H8A	0.9700
O3—C10'	1.450 (10)	C8—H8B	0.9700
O4—C9	1.383 (13)	C9—H9	0.9800
O4—C11	1.442 (10)	C10—C11	1.528 (10)
O4—C11'	1.454 (10)	C10—H10A	0.9700
O5—C12	1.173 (10)	C10—H10B	0.9700
O6—C13	1.201 (10)	C11—H11A	0.9700
O7—C20	1.398 (13)	C11—H11B	0.9700
O7—C21'	1.444 (9)	C10'—C11'	1.529 (10)
O7—C21	1.447 (10)	C10'—H10C	0.9700
O8—C20	1.396 (12)	C10'—H10D	0.9700
O8—C22'	1.443 (10)	C11'—H11C	0.9700
O8—C22	1.446 (10)	C11'—H11D	0.9700
N1—C2	1.386 (12)	C12—C15	1.479 (12)
N1—C1	1.417 (11)	C14—C15	1.342 (11)
N1—C6	1.467 (13)	C16—H16	0.9300
N2—C3	1.349 (11)	C17—H17A	0.9600
N2—C2	1.382 (12)	C17—H17B	0.9600
N2—C7	1.473 (12)	C17—H17C	0.9600
N3—C5	1.302 (11)	C18—H18A	0.9600
N3—C4	1.384 (11)	C18—H18B	0.9600
N3—C8	1.477 (11)	C18—H18C	0.9600
N4—C5	1.316 (10)	C19—C20	1.494 (14)
N4—C3	1.372 (11)	C19—H19A	0.9700
N4—H4	0.8600	C19—H19B	0.9700
N5—C13	1.344 (11)	C20—H20	0.9800
N5—C12	1.427 (11)	C22—C21	1.530 (10)
N5—C17	1.486 (13)	C22—H22A	0.9700
N6—C14	1.346 (11)	C22—H22B	0.9700
N6—C13	1.394 (13)	C21—H21A	0.9700
N6—C18	1.473 (12)	C21—H21B	0.9700
N7—C16	1.274 (11)	C22'—C21'	1.528 (10)
N7—C15	1.399 (10)	C22'—H22C	0.9700
N7—C19	1.456 (10)	C22'—H22D	0.9700
N8—C16	1.335 (11)	C21'—H21C	0.9700
N8—C14	1.365 (11)	C21'—H21D	0.9700
			
Cl1—Fe1—Cl3	111.77 (15)	O3—C10—C11	99.2 (16)
Cl1—Fe1—Cl2	109.10 (16)	O3—C10—H10A	111.9
Cl3—Fe1—Cl2	110.66 (17)	C11—C10—H10A	111.9
Cl1—Fe1—Cl4	109.65 (16)	O3—C10—H10B	111.9
Cl3—Fe1—Cl4	108.16 (16)	C11—C10—H10B	111.9
Cl2—Fe1—Cl4	107.41 (16)	H10A—C10—H10B	109.6
Cl8—Fe2—Cl6	109.43 (17)	O4—C11—C10	97.2 (16)
Cl8—Fe2—Cl5	109.77 (15)	O4—C11—H11A	112.3
Cl6—Fe2—Cl5	109.54 (15)	C10—C11—H11A	112.3
Cl8—Fe2—Cl7	110.21 (18)	O4—C11—H11B	112.3
Cl6—Fe2—Cl7	109.58 (17)	C10—C11—H11B	112.3
Cl5—Fe2—Cl7	108.28 (15)	H11A—C11—H11B	109.9
C9—O3—C10	100.2 (14)	O3—C10'—C11'	101.4 (17)
C9—O3—C10'	108.8 (12)	O3—C10'—H10C	111.5
C10—O3—C10'	25.3 (10)	C11'—C10'—H10C	111.5
C9—O4—C11	107.1 (13)	O3—C10'—H10D	111.5
C9—O4—C11'	103.8 (15)	C11'—C10'—H10D	111.5
C11—O4—C11'	27.7 (10)	H10C—C10'—H10D	109.3
C20—O7—C21'	112.1 (10)	O4—C11'—C10'	101.1 (15)
C20—O7—C21	106.0 (16)	O4—C11'—H11C	111.6
C21'—O7—C21	30.3 (14)	C10'—C11'—H11C	111.6
C20—O8—C22'	102.7 (12)	O4—C11'—H11D	111.6
C20—O8—C22	108.4 (14)	C10'—C11'—H11D	111.6
C22'—O8—C22	18.9 (19)	H11C—C11'—H11D	109.4
C2—N1—C1	128.4 (8)	O5—C12—N5	125.8 (9)
C2—N1—C6	115.5 (8)	O5—C12—C15	126.6 (8)
C1—N1—C6	116.1 (8)	N5—C12—C15	107.5 (8)
C3—N2—C2	119.4 (7)	O6—C13—N5	123.8 (10)
C3—N2—C7	121.2 (8)	O6—C13—N6	119.9 (8)
C2—N2—C7	119.3 (8)	N5—C13—N6	116.3 (7)
C5—N3—C4	107.5 (7)	C15—C14—N6	123.4 (7)
C5—N3—C8	125.4 (7)	C15—C14—N8	108.4 (7)
C4—N3—C8	126.9 (7)	N6—C14—N8	128.2 (7)
C5—N4—C3	107.2 (7)	C14—C15—N7	106.5 (7)
C5—N4—H4	126.4	C14—C15—C12	122.6 (8)
C3—N4—H4	126.4	N7—C15—C12	130.2 (8)
C13—N5—C12	130.1 (8)	N7—C16—N8	112.6 (8)
C13—N5—C17	115.8 (8)	N7—C16—H16	123.7
C12—N5—C17	114.1 (8)	N8—C16—H16	123.7
C14—N6—C13	119.3 (7)	N5—C17—H17A	109.5
C14—N6—C18	122.5 (8)	N5—C17—H17B	109.5
C13—N6—C18	118.1 (8)	H17A—C17—H17B	109.5
C16—N7—C15	106.8 (7)	N5—C17—H17C	109.5
C16—N7—C19	126.5 (7)	H17A—C17—H17C	109.5
C15—N7—C19	126.7 (7)	H17B—C17—H17C	109.5
C16—N8—C14	105.6 (7)	N6—C18—H18A	109.5
C16—N8—H8	127.2	N6—C18—H18B	109.5
C14—N8—H8	127.2	H18A—C18—H18B	109.5
O1—C1—N1	122.0 (9)	N6—C18—H18C	109.5
O1—C1—C4	128.4 (9)	H18A—C18—H18C	109.5
N1—C1—C4	109.5 (8)	H18B—C18—H18C	109.5
O2—C2—N2	120.6 (9)	N7—C19—C20	111.3 (8)
O2—C2—N1	123.2 (9)	N7—C19—H19A	109.4
N2—C2—N1	116.2 (8)	C20—C19—H19A	109.4
N2—C3—C4	123.5 (8)	N7—C19—H19B	109.4
N2—C3—N4	129.1 (7)	C20—C19—H19B	109.4
C4—C3—N4	107.4 (7)	H19A—C19—H19B	108.0
C3—C4—N3	106.5 (8)	O8—C20—O7	105.9 (8)
C3—C4—C1	122.3 (8)	O8—C20—C19	110.7 (9)
N3—C4—C1	130.6 (8)	O7—C20—C19	110.6 (8)
N3—C5—N4	111.3 (8)	O8—C20—H20	109.8
N3—C5—H5	124.3	O7—C20—H20	109.8
N4—C5—H5	124.3	C19—C20—H20	109.8
N1—C6—H6A	109.5	O8—C22—C21	103.2 (18)
N1—C6—H6B	109.5	O8—C22—H22A	111.1
H6A—C6—H6B	109.5	C21—C22—H22A	111.1
N1—C6—H6C	109.5	O8—C22—H22B	111.1
H6A—C6—H6C	109.5	C21—C22—H22B	111.1
H6B—C6—H6C	109.5	H22A—C22—H22B	109.1
N2—C7—H7A	109.5	O7—C21—C22	98.3 (18)
N2—C7—H7B	109.5	O7—C21—H21A	112.1
H7A—C7—H7B	109.5	C22—C21—H21A	112.1
N2—C7—H7C	109.5	O7—C21—H21B	112.1
H7A—C7—H7C	109.5	C22—C21—H21B	112.1
H7B—C7—H7C	109.5	H21A—C21—H21B	109.7
N3—C8—C9	110.9 (8)	O8—C22'—C21'	103.0 (13)
N3—C8—H8A	109.5	O8—C22'—H22C	111.2
C9—C8—H8A	109.5	C21'—C22'—H22C	111.2
N3—C8—H8B	109.5	O8—C22'—H22D	111.2
C9—C8—H8B	109.5	C21'—C22'—H22D	111.2
H8A—C8—H8B	108.0	H22C—C22'—H22D	109.1
O3—C9—O4	109.3 (10)	O7—C21'—C22'	96.4 (14)
O3—C9—C8	111.1 (8)	O7—C21'—H21C	112.5
O4—C9—C8	109.3 (9)	C22'—C21'—H21C	112.5
O3—C9—H9	109.0	O7—C21'—H21D	112.5
O4—C9—H9	109.0	C22'—C21'—H21D	112.5
C8—C9—H9	109.0	H21C—C21'—H21D	110.0

### Hydrogen-bond geometry (Å, °)

**Table:**

*D*—H···*A*	*D*—H	H···*A*	*D*···*A*	*D*—H···*A*
N4—H4···O2^i^	0.86	1.93	2.774 (9)	165
N8—H8···O6^ii^	0.86	1.91	2.754 (9)	166
C5—H5···Cl5^i^	0.93	2.80	3.650 (11)	153
C8—H8B···O7^iii^	0.97	2.29	3.114 (11)	142
C10—H10C···O5^iv^	0.95	2.46	3.098 (11)	124
C10'—H10B···O5^iv^	0.89	2.57	3.384 (11)	153
C10'—H10C···O5^iv^	0.97	2.46	3.384 (11)	159
C16—H16···Cl1^iii^	0.93	2.78	3.560 (11)	143
C19—H19A···O4^v^	0.97	2.48	3.227 (11)	134
C19—H19A···O5	0.97	2.53	3.167 (11)	123
C22'—H22D···O1	0.97	2.58	3.242 (11)	126

Symmetry codes: (i) −*y*+1, *x*, *z*+1/2; (ii) *y*, −*x*, *z*+1/2; (iii) −*x*+1, −*y*, *z*; (iv) *x*, *y*, *z*+1; (v) −*x*+1, −*y*, *z*−1.

## References

[bb1] Barbaro, P., Bianchini, C., Fochi, M., Masi, D. & Mealli, C. (1992). *Acta Cryst.* **C48**, 625–627.

[bb2] Bottomley, G. A., Carter, A. M., Engelhardt, L. M., Lincoln, F. J. & Patric, J. M. (1984). *Aust. J. Chem.* **37**, 871–877.

[bb3] Bruker (2000). *SMART*, *SADABS* and *SAINT* Bruker AXS Inc., Madison, Wisconsin, USA.

[bb4] Flack, H. D. (1983). *Acta Cryst.* A**39**, 876–881.

[bb5] Franzone, J. S., Cirillo, R. & Biffignandi, P. (1989). *Eur. J. Pharmacol.* **165**, 269–274. 10.1016/0014-2999(89)90721-8 2776832

[bb6] Franzone, J. S., Reboani, C. & Fonzo, D. (1981). *Farmacol. Sci.* **36**, 201–219. 6262105

[bb7] Li, C. H., Luo, Z. & Li, Z. H. (1995). *Chin. J. Pharm.* **25**, 385–386.

[bb8] Sheldrick, G. M. (2008). *Acta Cryst.* A**64**, 112–122. 10.1107/S0108767307043930 18156677

[bb9] Zhao, J. J. & Li, L. (2001). *J. Norman Bethune Univ. Med. Sci.* **27**, 646–676.

